# Patient-reported outcome labeling claims and measurement approach for metastatic castration-resistant prostate cancer treatments in the United States and European Union

**DOI:** 10.1186/s12955-014-0104-5

**Published:** 2014-07-04

**Authors:** Marci J Clark, Nimanee Harris, Ingolf Griebsch, Dagmar Kaschinski, Catherine Copley-Merriman

**Affiliations:** 1 RTI Health Solutions, 3005 Boardwalk Drive, Suite 105, Ann Arbor 48108, MI, USA; 2 Boehringer Ingelheim GmbH, Binger Straße 173/3460-04-03, Ingelheim D-55216, Germany

**Keywords:** Patient-reported outcomes, Questionnaires, Metastatic castration-resistant prostate cancer, Treatment, Labeling, Food and drug administration, European medicines agency

## Abstract

**Background:**

Metastatic castration-resistant prostate cancer (mCRPC) and its treatment significantly affect health-related quality of life (HRQOL). Our objectives were to evaluate and compare patient-reported outcome (PRO) claims granted by the Food and Drug Administration (FDA) and European Medicines Agency (EMA) for 5 recently approved mCRPC treatments and to examine key characteristics, development, and measurement properties of the PRO measures supporting these claims against current regulatory standards.

**Methods:**

Five products approved for treatment of mCRPC by the FDA and the EMA (2010–2013) were examined: enzalutamide, abiraterone, sipuleucel-T, cabazitaxel, and radium Ra 223 dichloride. United States (US) drug approval packages and European Public Assessment Reports were reviewed. PRO claims in the US labels and European Summaries of Product Characteristics and supporting measures were identified. For PRO measures supporting claims, a targeted literature review was conducted to identify information on key characteristics and measurement properties; this information was compared against FDA PRO guidance criteria.

**Results:**

Nine PRO “claims” were granted across 4 of 5 products reviewed. The EMA granted more claims (7 claims—4 for pain, 3 for HRQOL) than the FDA (2 claims, both for pain). The Brief Pain Inventory–Short Form (BPI-SF) worst pain item supported most pain claims and was the only measure supporting US claims. EMA pain claims were supported by BPI-SF worst pain (n = 2) and average pain (n = 1) items and the McGill Pain Questionnaire Present Pain Intensity component (n = 1). EMA HRQOL claims were supported by the Functional Assessment of Cancer Therapy–Prostate Module (n = 2) and the EuroQol 5 Dimensions with visual analogue scale (n = 1). Pain and prostate cancer–specific HRQOL measures supporting claims met US regulatory standards for construct validity, reliability, and responsiveness; these properties were strongest for the BPI-SF worst pain item. Only the BPI-SF worst pain item has documented content validity in mCRPC.

**Conclusions:**

PRO label claims were commonly granted across the mCRPC products reviewed. Among the measures reviewed, only the BPI-SF worst pain item supported US label claims. The BPI-SF worst pain item is recommended for pain assessment for the evaluation of new mCRPC treatments.

## Background

Prostate cancer (PC) is the most commonly diagnosed solid organ malignancy in the United States (US) and the second-leading cause of cancer deaths among American men [[1]]. These deaths are often the result of metastatic castration-resistant prostate cancer (mCRPC), which historically has had a median survival of fewer than 2 years [[1]].

More than 90% of patients with mCRPC develop bone metastases [[2]]. Bone involvement in mCRPC can lead to significant morbidity, which includes pain and skeletal-related events such as spinal cord compression, pathological fractures, hypercalcemia of malignancy, requirement for interventions such as bone surgery, or need for bone radiation [[2]]. Other symptoms of mCRPC include anorexia, anxiety, constipation, diarrhea, sleep disturbance, mucositis, nausea, peripheral sensory neuropathy, rash, vomiting, and urinary symptoms [[3]]. Fatigue is another dominant PC symptom and the most common adverse event resulting from mCRPC treatment [[4]]. These disease symptoms and noted side effects of treatment can significantly affect health-related quality of life (HRQOL). Therefore, patient-reported outcomes (PROs) are important to assess in patients with mCRPC to develop an understanding of the patient-subjective experience with disease and treatment; they are also particularly important when new treatments have a modest impact on survival [[3],[5]]. In addition, a recent study by Miller and colleagues found that achieving dual PRO claims in the US and the European Union (EU) has led to increased market share for oncology products in the US [[6]].

In 2004, two pivotal trials, TAX-327 and SWOG 9916, supported the approval of docetaxel as the first mCRPC treatment, in combination with prednisone, found to prolong median overall survival by approximately 3 months when compared with the combination of mitoxantrone and prednisone [[7],[8]]. Consequently, docetaxel became the standard first-line regimen in patients with mCRPC [[9]]. A variety of chemotherapies, targeted therapies, and immunotherapies since have been developed and approved for use in mCRPC, with the goal of improved efficacy outcomes when compared with docetaxel [[9]]. Zytiga (abiraterone), Jevtana (cabazitaxel), Xtandi (enzalutamide), Xofigo (radium Ra 223 dichloride), and Provenge (sipuleucel-T) were recently approved (2010–2013) by both the US Food and Drug Administration (FDA) and the European Medicines Agency (EMA) for the treatment of mCRPC. In addition to survival, PRO endpoints were included in clinical registration trials for each of these newly approved products to assess HRQOL and other concepts of importance to patients with mCRPC.

The objectives of this research were to evaluate and compare PRO label claims granted by the FDA and the EMA for these five recently approved mCRPC treatments and to examine the key characteristics, development, and measurement properties of the PRO instruments for which label claims were achieved against current regulatory standards for PROs [[10]]. The five recently approved drugs were chosen because they reflect the most current regulatory thinking. The research findings can be used to inform the development of a PRO strategy for phase 2 and 3 clinical trials designed to support a product label claim for a new treatment for mCRPC.

## Methods

### Identification of PRO label claims and supporting measures

The website Drugs@FDA was searched to identify the latest product label and drug approval package (DAP) for abiraterone, cabazitaxel, enzalutamide, radium Ra 223 dichloride, and sipuleucel-T [[11]]. Relevant information was retrieved from the Medical Review section of each DAP. In addition, European Public Assessment Report (EPAR), including the most recent version of the Summary of Product Characteristics (SmPC) and scientific discussion documents, were identified for each product using the EMA website [[12]].

The labels and SmPCs were reviewed to identify PRO label claims and the measures supporting them. DAPs and EPARs were reviewed to determine any PRO measures that resulted in PRO claims but were not specifically named in the label or SmPC. Any indication of PRO-related comments by the reviewing division or the Study Endpoints and Label Development (SEALD) group were also noted when reviewing US DAPs and labels.

For analysis purposes, a PRO claim was defined as any mention of a PRO measure or patient-reported endpoint (whether or not the PRO measure was named; e.g., “proportion of patients with pain palliation”) anywhere in the product label or SmPC. A product with multiple analyses and results described in the label or SmPC based on the same PRO measure counted as a single claim. Each product may have had more than one PRO claim. PRO label claims were classified based on the following types: symptom (pain), PC-specific HRQOL, and generic HRQOL. Symptom-specific claims based on PRO measures focusing solely on assessment of the concept of pain were classified as “symptom (pain).” HRQOL is a multidimensional concept that represents the patient’s general perception of the effect of illness and treatment on physical, psychological, and social aspects of life [[10]]. “Generic HRQOL” claims were considered to be based on PRO measures assessing HRQOL not specific to any particular disease. “PC-specific HRQOL” claims were defined as being based on measures focused on assessment of the impact of PC on HRQOL.

Frequencies and cross-tabulation of label claim types were calculated using Microsoft Excel 2010 (Excel). PRO label claims and the measures supporting them were also summarized descriptively in tabular format.

### Evaluation of selected PRO measures

For select PRO measures achieving label claims, targeted searches of PubMed/Medline (back to 2003) were conducted to identify relevant articles for review based on abstracts in English. Additional desktop searches of instrument websites, the public-access version of PROQOLID, and an internal PRO instrument repository at RTI Health Solutions (RTI-HS) were also conducted to identify relevant information and references, especially those published prior to 2003 [[13]].

The primary goal of these searches was to identify relevant information sources or publications for individual PRO measures, as available, describing instrument development and psychometric properties (validity, reliability, and responsiveness) in patients with mCRPC and information on how to determine a clinically meaningful change. We also sought to identify key characteristics of each PRO measure supporting label claims including constructs/subscales, the number of items, response scales, and recall period. All relevant information identified was recorded in tabular format in Excel.

The following information concerning the development process was identified for each measure, as available, to inform the assessment of content validity: whether patients with mCRPC were included during the development process, whether input from clinicians or health care professionals (HCPs) treating patients with mCRPC was included during measure development, and whether a literature review was conducted to identify items of importance to patients with mCRPC during instrument development.

When extracting relevant information from the source documents for each measure of interest, content validity, internal consistency, test-retest reliability, construct and known-groups validity, and ability to detect change (also referred to as responsiveness) were defined as per the FDA PRO guidance [[10]].

Responsiveness is best assessed in the context of a therapeutic intervention that has been shown to be effective. Therefore, to confirm PRO measure responsiveness in patients with mCRPC, targeted searches were completed to identify any phase 2 or 3 randomized controlled trials evaluating treatments for mCRPC that included each relevant PRO measure, especially studies supporting an approved PRO label claim for an mCRPC product of interest. Clinical trials that included the PRO measures but did not evaluate one of the five mCRPC treatments of interest were excluded. Full-text articles were preferred, but abstracts with relevant information were also included. Two recent review articles were also searched to identify studies cited demonstrating relevant PRO measure responsiveness [[3],[9]].

Finally, published information was sought for each PRO measure of interest to identify recommendations for the amount of change required to justify a clinically meaningful change for an individual with mCRPC.

The available data identified regarding instrument characteristics, development, validation, responsiveness, and interpretation for each relevant PRO measure were reviewed against FDA PRO guidance criteria, the highest level of evidence currently required by a regulatory agency to support medical product labeling claims [[10]]. Key considerations in the evaluation of a PRO instrument’s adequacy to support claims in medical product labeling include satisfactory documented evidence of content validity and established measurement properties (i.e., reliability, validity, ability to detect change) in the target clinical trial population. The FDA reviews documentation of the PRO instrument development and evaluation in combination with clinical trial results to determine whether a labeling claim is substantiated [[10]]. Further critical aspects considered by the FDA regarding the appropriate incorporation of the PRO measure into the protocol and statistical analysis plan include, but are not limited to, plans for handling missing data, adjustment for multiplicity, and plans for interpretation beyond statistical significance.

The EMA has not released a formal guidance document related to PROs; instead, it has issued a reflections paper that provides broad recommendations regarding HRQOL evaluation, a specific type of PRO in the context of clinical trials to support claims [[14]]. Although there are many similarities in the requested information for HRQOL measures (e.g., documentation of validation in the target population; appropriate incorporation of the PRO measure into the protocol and statistical analysis plan including, but not limited to, plans for handling missing data, adjustment for multiplicity, and plans for interpretation beyond statistical significance), the level of documentation is considerably less than that required by the FDA PRO guidance [[10]].

## Results

### Identification of PRO label claims and supporting measures

Four of the five mCRPC products reviewed received one or more PRO label claims (Table 1). Only sipuleucel-T was not granted a PRO label claim by either the FDA or the EMA.

**Table 1:** PRO label claims achieved in the US compared to the EU

**Product (brand name/generic name)**	**US approval year**	**US product label claim(s)**	**EU approval year**	**EU SmPC claim(s)**
Xtandi/enzalutamide	2012	Yes	2013	Yes
Zytiga/abiraterone	2011	Yes	2011	Yes
Jevtana/cabazitaxel	2010	No	2011	Yes
Xofigo/radium Ra 223 dichloride	2013	No	2013	Yes
Provenge/sipuleucel-T	2010	No	2013	No

EU = European Union; SmPC = Summary of Product Characteristics; US = United States.

Table 2 summarizes the type of PRO claim granted by the EMA or the FDA for the products reviewed. A total of nine PRO claims were granted across the four mCRPC products that achieved claims. Pain claims were the most common (n = 6), followed by HRQOL claims (n = 3 [PC-specific HRQOL, n = 2; generic HRQOL, n = 1]). The EMA granted more PRO claims (n = 7) than the FDA (n = 2) among the four reviewed products with claims. The FDA granted only pain claims for two products, enzalutamide and abiraterone. The EMA granted pain claims (n = 4) for enzalutamide, abiraterone, and cabazitaxel and granted HRQOL claims (n = 3) for abiraterone and Ra-223.

**Table 2:** PRO claim types granted by the FDA or the EMA

**Type of claim**	**FDA-granted claim (N = 5 products)**	**EMA-granted claim (N = 5 products)**	**Total claims (N = 5 products)**
Symptom: pain	2	4	6
Prostate cancer–specific HRQOL	0	2	2
Generic HRQOL	0	1	1
**Total claims**	**2**	**7**	**9**

EMA = European Medicines Agency; FDA = Food and Drug Administration; HRQOL = health-related quality of life.

As shown in Table 3, the Brief Pain Inventory–Short Form (BPI-SF) worst pain item supported the majority of the pain claims and was the only measure supporting US PRO claims. The EMA-approved pain claims were supported by the BPI-SF worst pain item (n = 2), the BPI-SF average pain item (n = 1), and the Present Pain Intensity (PPI) component of the McGill Pain Questionnaire (MPQ) (n = 1). HRQOL measures supporting EMA-approved claims included a PC-specific measure, the Functional Assessment of Cancer Therapy–Prostate Module (FACT-P) (n = 2), and a generic measure, the EuroQol 5 Dimensions (EQ-5D) questionnaire with visual analogue scale (VAS) (n = 1).

**Table 3:** PRO measures supporting label claims in the US and EU

**Product (brand name name/generic name)**	**PRO measure(s) supporting US product label claim(s)**	**PRO measure(s) supporting EU SmPC claim(s)**
Xtandi/enzalutamide	BPI-SF worst pain item^a^	BPI-SF worst pain item^a^
Zytiga/abiraterone	BPI-SF worst pain item	BPI-SF worst pain and average pain intensity items; FACT-P
Jevtana/cabazitaxel	None	PPI scale from the McGill-Melzack questionnaire^b^
Xofigo/radium Ra 223 dichloride	None	FACT-P
		EQ-5D with VAS
Provenge/sipuleucel-T	None	None

BPI-SF = Brief Pain Inventory–Short Form; EU = European Union; EQ-5D with VAS = EuroQoL 5 Dimensions with visual analogue scale; FACT-P = Functional Assessment of Cancer Therapy–Prostate Module; PPI = Present Pain Intensity; PRO = patient-reported outcome; SmPC = Summary of Product Characteristics; US = United States.

^a^The BPI was described in four different ways between the US label and EU SmPC for Xtandi (i.e., BPI, BPI-SF, BPI worst pain item and BPI-SF worst pain item). For the purposes of PRO label claim analyses and given other consistencies between descriptions, we assumed the BPI-SF worst pain item was actually implemented in the Xtandi studies for both the US and EU.

^b^The McGill-Melzack questionnaire is also known as the McGill Pain Questionnaire (MPQ).

Table 4 summarizes the PRO claim language granted by the FDA and the EMA across the four mCRPC products with claims. All the PRO claims were identified in the Clinical Studies section of the US labels and Clinical Efficacy and Safety section of the EU SmPCs. The PRO claims for enzalutamide in both the US and the EU were limited to the description of baseline data for the BPI-SF worst pain item. The only PRO claim in the US indicating positive data was the “time to opiate use result was supported by a delay in patient reported pain progression” for abiraterone, based on the BPI-SF worst pain item. No other PRO claims were permitted in the US for the products reviewed.

**Table 4:** PRO claims granted by the FDA and the EMA

**Product (brand name/generic name)**	**US product label claim(s)**	**EU SmPC Claim(s)**
Xtandi/enzalutamide	“Baseline data: 28% had a mean Brief Pain Inventory score of ≥ 4”	“28.4% had a mean Brief Pain Inventory score of ≥ 4 (mean of patient’s reported worst pain over the previous 24 hours calculated for seven days prior to randomization).”
	“P-value is derived from a log-rank test stratified by baseline ECOG performance status score (0–1 vs. 2) and mean baseline pain score (BPI-SF score < 4 vs. ≥ 4)”	Overall survival presented in a table by subgroup; one of the subgroups was “Baseline mean pain score on BPI-SF Question #3^a^” of < 4 compared to ≥ 4
Zytiga/abiraterone	Study 1 (baseline data):	Study 302:
	“45% had a Brief Pain Inventory-Short Form score of ≥ 4 (patient’s reported worst pain over the previous 24 hours)”	“A score of 0–1 on Brief Pain Inventory-Short Form (BPI-SF) worst pain in last 24 hours was considered symptomatic, and a score of 2–3 was considered mildly symptomatic.”
	Study 2:	
	“Baseline pain assessment was 0–1 (asymptomatic) in 66% of patients and 2–3 (mildly symptomatic) in 26% of patients as defined by the Brief Pain Inventory-Short Form (worst pain over the last 24 hours).”	“Pain: Treatment with ZYTIGA significantly reduced the risk of average pain intensity progression by 18% compared with placebo (p = 0.0490). The median time to progression was 26.7 months in the ZYTIGA group and 18.4 months in the placebo group.”
	“The median time to opiate use for prostate cancer pain was not reached for patients receiving ZYTIGA and was 23.7 months for patients receiving placebo (HR = 0.686; 95% CI: [0.566, 0.833], p = 0.0001). The time to opiate use result was supported by a delay in patient reported pain progression favoring the ZYTIGA arm.”	“Time to degradation in the FACT-P (Total Score): Treatment with ZYTIGA decreased the risk of FACT-P (Total Score) degradation by 22% compared with placebo (p = 0.0028). The median time to degradation in FACT-P (Total Score) was 12.7 months in the ZYTIGA group and 8.3 months in the placebo group.”
		Study 301:
		“The proportion of patients with pain palliation was statistically significantly higher in the ZYTIGA group than in the placebo group (44% versus 27%, p = 0.0002). A responder for pain palliation was defined as a patient who experienced at least a 30% reduction from baseline in the BPI-SF worst pain intensity score over the last 24 hours without any increase in analgesic usage score observed at two consecutive evaluations four weeks apart. Only patients with a baseline pain score of ≥ 4 and at least one post-baseline pain score were analysed (N = 512) for pain palliation.”
		“A lower proportion of patients treated with ZYTIGA had pain progression compared to patients taking placebo at 6 (22% versus 28%), 12 (30% versus 38%) and 18 months (35% versus 46%). Pain progression was defined as an increase from baseline of ≥ 30% in the BPI-SF worst pain intensity score over the previous 24 hours without a decrease in analgesic usage score observed at two consecutive visits, or an increase of ≥ 30% in analgesic usage score observed at two consecutive visits.”
		“The time to pain progression at the 25th percentile was 7.4 months in the ZYTIGA group, versus 4.7 months in the placebo group.”
Jevtana/cabazitaxel	None	“Secondary endpoints include pain progression [assessed using the Present Pain Intensity (PPI) scale from the McGill-Melzack questionnaire and an Analgesic Score (AS)] and pain response (defined as 2-point greater reduction from baseline median PPI with no concomitant increase in AS, or reduction of ≥ 50% in analgesic use from baseline mean AS with no concomitant increase in pain).”
		“There was no statistical difference between both treatment arms in pain progression and pain response.”
Xofigo/radium Ra 223 dichloride	None	“Health Related Quality of Life (HRQOL) was assessed in the phase III ALSYMPCA study using specific questionnaires: the EQ-5D (generic instrument) and the FACT-P (prostate cancer specific instrument). Both groups experience a loss of quality of life. Relative to placebo, the decline in quality of life was slower for Xofigo during the on-treatment period as measured by EQ-5D utility index score (−0.040 versus −0.109; p = 0.001), EQ-5D self-reported Visual Analogue health status scores (VAS) (−2.661 versus −5.860; p = 0.018) and the FACT P total score (−3.880 versus −7.651, p = 0.006) but did not reach published minimally important differences. There is limited evidence that the delay in loss of HRQOL extends beyond the treatment period.”
		“The results from the phase III ALSYMPCA study regarding time to external beam radiation therapy (EBRT) for pain relief and fewer patients reporting bone pain as an adverse event in the Xofigo group indicate a positive effect on bone pain.”

BPI-SF = Brief Pain Inventory–Short Form; CI = confidence interval; ECOG = Eastern Cooperative Oncology Group; EQ-5D = EuroQol 5 Dimensions; EU = European Union; FACT-P = Functional Assessment of Cancer Therapy–Prostate Module; FDA = Food and Drug Administration; EMA = European Medicines Agency; HR = hazard ratio; HRQOL = health-related quality of life; SmPC = Summary of Product Characteristics; US = United States.

^a^BPI-SF Question #3 asks about worst pain in the last 24 hours.

EU claims related to pain progression and pain response were common to both abiraterone and cabazitaxel SmPCs. The abiraterone SmPC had multiple results described based on positive data from both the BPI-SF worst pain item (including median time to pain progression [months], proportion [%] of patients with pain palliation, proportion [%] of patients with pain progression, and time [months] to pain progression at the 25th percentile) and the BPI-SF average pain item (reduction in risk [%] of average pain intensity progression). In contrast, the cabazitaxel SmPC indicated that “there was no statistical difference between both treatment arms in pain progression and pain response” based on the PPI scale from the MPQ. The abiraterone and radium Ra 223 dichloride SmPCs both included HRQOL claims based on the FACT-P. Abiraterone’s claims were based on both the risk (%) of and median time (months) to FACT-P total score degradation, whereas radium Ra 223 dichloride’s claim was based on changes from baseline in FACT-P total score. The radium Ra 223 dichloride SmPC also included claim language based on changes from baseline in the EQ-5D and VAS.

### Evaluation of instrument properties for selected PRO measures against FDA guidance criteria

Two targeted searches were conducted in PubMed/Medline: one search to identify literature describing the key instrument characteristics, development, and validation information for the BPI-SF worst pain item, BPI-SF average pain item, MPQ PPI, and FACT-P; and the other search to identify literature describing the responsiveness of these measures in clinical registration trials for the five mCRPC treatments of interest.

The two PubMed/Medline searches identified a total of 71 articles for review; of these, 65 were excluded because they did not contain relevant information, and 6 were included. Additionally, two papers cited in the review articles examined, two papers obtained from the RTI-HS PRO instrument repository, and one paper identified on an instrument developer’s website were included. Eleven papers, in total, were reviewed and are summarized. One published abstract was identified, and the poster with more extensive information, which was provided by the authors upon request, is summarized. The EuroQoL website also provided relevant information for inclusion in this paper. Finally, key measurement characteristics (e.g., number of items, recall period) for the four measures were pulled from PROQOLID to supplement information found in the papers reviewed. The information identified for the four PRO measures of interest then was evaluated against FDA guidance criteria [[10]] for PRO measures intended to support labeling claims in the US. The EQ-5D supported a PRO claim in the SmPC for radium Ra 223 dichloride but was excluded from review and evaluation because it is a generic HRQOL measure used broadly in most therapeutic areas to determine health utility scores for economic modeling [[15]].

Table 5 summarizes the key characteristics and content validity for the PRO measures reviewed.

**Table 5:** Key characteristics and instrument development evidence supporting content validity

	**BPI-SF worst pain item**	**BPI-SF average pain item**	**PPI (from MPQ)**	**FACT-P**
**Key characteristics**				
Type of measure	Symptom: pain	Symptom: pain	Symptom: pain	PC-specific HRQOL
Constructs/subscales	Pain intensity (or severity): worst pain^a^	Pain intensity (or severity) average pain	Present pain intensity	FACT-G subscales:
				▪ Physical well-being
				▪ Social well-being
				▪ Emotional well-being
				▪ Functional well-being
				Plus PC subscale (12 site-specific items to assess prostate related “additional concerns”)
Number of items	1	1	1	39 (27 FACT-G; 12 PC specific)
Recall period	Past 24 hours	Unspecified	Present	Past 7 days
Response scale	0-10 NRS	0-10 NRS	0-6 VRS	5-point Likert scale
**Instrument development: support for content validity**				
Patients with mCRPC included in development process	–/✓^a^	–^a^	–^a^	–/✓^b^
Input from clinicians or HCPs treating patients with mCRPC included in development measure	–	–	–	–
Items of importance to patients with mCRPC identified from literature review during instrument development	–	–	–	–

BPI-SF = Brief Pain Inventory–Short Form; FACT-G = Functional Assessment of Cancer Therapy–General; FACT-P = Functional Assessment of Cancer Therapy—Prostate Module; HCP = health care professional; HRQOL = health-related quality of life; mCRPC = metastatic castration-resistant prostate cancer; MPQ = McGill Pain Questionnaire; NRS = numeric rating scale; PC = prostate cancer; PPI (from MPQ) = Present Pain Intensity component of the McGill Pain Questionnaire; VRS = verbal rating scale.

✓= Yes, evidence identified; − = No evidence identified.

^a^Gater and colleagues evaluated the content validity of the BPI-SF “average pain” and “worst pain” items (both assessed on a 0–10 numeric rating scale) in cognitive debriefing interviews with 17 patients with mCRPC [[21]]. Results strongly supported content validity for the “worst pain item” in this population. However, there was variability in patients’ interpretation of the “average pain” item, so results did not support the content validity of this item in patients with mCRPC. The MPQ PPI (assessed on a 0–6 VRS) was also evaluated. Patients’ interpretation of the PPI item was variable, and they also had difficulty with the VRS [[21]].

^b^According to Esper and colleagues, eight individuals with PC contributed to item development, and 25 individuals with PC at various stages and 10 additional patients with PC who had undergone radical prostatectomy completed and provided feedback on the first draft of the FACT-P [[18]]. However, the exact number of patients with mCRPC included during development was not reported. Based on a personal communication with the instrument developer, Dr. David Cella, most patients participating in the Esper study were men with early stage disease and only some had advanced, metastatic and castrate-resistant disease [[18]].

#### Key instrument characteristics

The BPI-SF is a pain-specific measure developed by Charles Cleeland to assess patient-reported severity (or intensity) of pain (4 items) and impact of pain on daily functioning (11 items) in patients with cancer pain [[16]]. Two of the items on the BPI-SF pain severity subscale assess worst pain in the past 24 hours and average pain (recall period not specified), respectively. For all items on this subscale, patients are asked to rate their pain on an 11-point numeric rating scale with anchors of 0 (no pain) and 10 (pain as bad as you can imagine). The worst pain item’s brief recall period is favored by the FDA, whereas the average pain item does not specify a recall period and may be considered less favorable.

The PPI is a single item developed by Ronald Melzack to measure patient-reported pain intensity with present recall. The PPI is one component and an individually scored subscale of the 20-item MPQ [[17]]. The PPI asks patients to rate their pain using a 6-point verbal rating scale (0 = No pain, 1 = Mild, 2 = Discomforting, 3 = Distressing, 4 = Horrible, 5 = Excruciating). The PPI is symptom-specific, brief, and has a short recall period, all considered favorable characteristics by the FDA [[10]].

The FACT-P is a 39-item questionnaire developed by Dr. David Cella and colleagues at Functional Assessment of Chronic Illness Therapy to assess patient-reported HRQOL and PC-specific symptoms [[18]]. The FACT-P consists of the 27-item Functional Assessment of Cancer Therapy–General (FACT-G) and a 12-item PC subscale. The 27 items in the FACT-G are grouped into four domains: physical well-being, social/family well-being, emotional well-being, and functional well-being. The PC subscale assesses pain (3 items), urination problems (3 items), and sexual functions (2 items). In addition, it contains items for weight loss, appetite, overall comfort, and bowel movement. FACT-P items ask patients to recall over the past 7 days and are rated using a 5-point Likert rating scale (0 = Not at all; 1 = A little bit; 2 = Somewhat; 3 = Quite a bit; and 4 = Very much). Although multi-item scales assessing HRQOL have been accepted for labeling since the release of the draft PRO guidance by the FDA in 2006 (e.g., Soliris [eculizumab] and Letairis [ambrisentan]), brief measures examining disease symptoms or functioning with short recall periods are preferred by the FDA, and the majority of US label claims have been based on these types of PRO measures [[10],[19],[20]].

#### Instrument development and content validity

Given the emphasis of the FDA PRO guidance on content validity, detailed information on the development of each PRO measure of interest was sought to inform the assessment of each measure’s content validity in patients with mCRPC [[10]]. Although patients with mCRPC were not included during initial development of the BPI-SF or MPQ, Gater and colleagues evaluated the content validity of the BPI-SF “worst pain” and “average pain” items (both assessed on a 0–10 numeric rating scale) as well as the MPQ PPI (assessed on a 0–6 verbal rating scale) in cognitive debriefing interviews with 17 patients with mCRPC [[21]]. Results of this study strongly supported content validity for the “worst pain item,” whereas the results did not support content validity for the “average pain” item or the MPQ PPI item. Esper and colleagues reported that a total of 43 individuals with PC were involved in the development of the FACT-P [[18]]. Although the exact number of patients with mCRPC included during development was not reported in the publication, Dr. David Cella, the FACT-P developer, indicated (personal communication, 2014) that most of the patients in the Esper study were men with early stage disease, although some had advanced, metastatic, and castration-resistant disease. No further published information is available on the content validity of the FACT-P in patients with mCRPC [[18]].

No evidence was identified that input from clinicians or HCPs treating patients with mCRPC was included in the development of any of the four measures reviewed, nor were items of importance to patients with mCRPC identified from the literature review during instrument development.

In summary, only the BPI-SF worst pain item has documented content validity in patients with mCRPC, consistent with FDA criteria.

#### Psychometric properties

Table 6 summarizes the published evidence of instrument reliability (internal consistency and test-retest reliability), construct validity (convergent/divergent), known-groups validity, responsiveness, and interpretation in patients with mCRPC for the four PRO measures reviewed.

**Table 6:** Psychometric properties

**Psychometric properties reported in published literature for patients with mCRPC**^**a**^	**BPI-SF worst pain item**	**BPI-SF average pain item**	**PPI item from MPQ**	**FACT-P**
Reliability				
Test-retest	✓	–/✓^b^	–/✓^c^	✓
Internal consistency	NA	NA	NA	✓
Construct validity				
Convergent	✓	✓	✓	✓^d^
Divergent	✓	✓	✓	✓^d^
Known-groups/discriminant validity	–	–	–	✓
Responsiveness^e^	✓	✓	–/✓^f^	**✓**
Interpretation/clinically meaningful change established	✓^g^	–	–	✓ ^g^
References	[[16],[23],[31],[34],[26],[27]]	[[16],[23],[34]]	[[17],[21],[23],[30]]	[[18],[23],[28],[32],[34],[26],[29]]

BPI-SF = Brief Pain Inventory–Short Form; FACT-P = Functional Assessment of Cancer Therapy–Prostate Module; ICC = intraclass correlation coefficient; mCRPC = metastatic castration-resistant prostate cancer; MPQ = McGill Pain Questionnaire; NA = not applicable; PPI (from MPQ) = Present Pain Intensity component of the McGill Pain Questionnaire.

^a^✓= Yes, measurement property identified as evaluated based on patients with mCRPC; − = No, measurement property not identified as evaluated based on patients with mCRPC.

^b^Robinson and colleagues reported acceptable (i.e., ICC > 0.70) test-retest reliability for the BPI-SF pain intensity scale and worst pain item alone but not for the individual average pain item [[23]].

^c^Robinson and colleagues reported nonacceptable (i.e., ICC < 0.70) test-retest reliability of ICC = 0.56 for the PPI in one trial of 69 patients with mCRPC but acceptable test-retest reliability (ICC = 0.85) in another trial of 93 patients with mCRPC [[23]].

^d^Convergent and divergent validity established for the four-item FACT-P pain scale, prostate cancer subscale, and total scale scores in patients with mCRPC [[23]].

^e^Evidence of responsiveness of the PRO measure in one or more phase 2 or 3 randomized controlled trials evaluating one of the mCRPC drugs of interest.

^f^Pain progression and pain response endpoints did not differentiate between treatment arms in the phase 3 registration study for cabazitaxel in patients with mCRPC [[30]].

^g^Patient scores ≥ 5 on the BPI-SF worst pain item are associated with significant and meaningful impairments in patients with mCRPC, thus supporting the adequacy of this cut point as an appropriate definition of pain progression in this population [[31]]; a clinically meaningful change of 6 to 10 was estimated for the FACT–P total score (score range: 0–156) [[32]].

### Reliability

Internal consistency was not applicable for assessment for the BPI-SF worst pain and average pain items or the PPI because these are single-item measures. Acceptable internal consistency (i.e., Cronbach’s alpha above 0.70 but not higher than 0.95) was reported for the FACT-P, with Cronbach’s alpha coefficients between 0.78 and 0.83 in patients with mCRPC [[22],[23]].

Test-retest reliability was reported as acceptable (i.e., intraclass correlation coefficient [ICC] of 0.70 or greater) for the BPI-SF worst pain item and FACT-P in two trials evaluating treatment for patients with mCRPC [[23],[24]]. Robinson and colleagues reported acceptable test-retest reliability for the BPI-SF pain intensity scale (all 4 items scored together; 0.73 and 0.90), but this information was not available for the individual average pain item [[23]].

### Construct and known-groups validity

A priori hypotheses were not identified to assess construct validity for any of the four PRO measures of interest in any study evaluating patients with mCRPC. Convergent validity was demonstrated for the BPI-SF worst pain and average pain items, PPI, and FACT-P primarily through Pearson correlations (*r*) between each of these instruments to assess similar constructs using data from two trials evaluating treatment for patients with mCRPC [[23]]. Correlation values were categorized as < 0.10, weak; 0.10-0.50, moderate; and > 0.50, strong [[25]]. Pearson correlations were strong between the BPI-SF worst pain and average pain (*r* = 0.79; *P* < 0.006) items; there was a moderate correlation between the BPI-SF worst pain item and the FACT-P total score (*r* = −0.42; *P* < 0.006) and a strong correlation with the PPI (*r* = 0.52; *P* < 0.006). The FACT-P total score was also strongly correlated with the BPI-SF average pain (*r* = 0.57; *P* < 0.006) item and moderately correlated with the PPI (*r* = 0.34; *P* < 0.006) [[23]]. No evidence of divergent validity or known-groups validity was identified for any of the four PRO measures of interest in patients with mCRPC.

### Ability to detect change (responsiveness)

Three of the four PRO measures of interest demonstrated responsiveness in one or more phase 3 randomized controlled trials evaluating recently approved treatment for patients with mCRPC (Table 6). The BPI-SF worst pain item was responsive in the published phase 3 registration studies supporting the approval of abiraterone for patients with mCRPC [[26],[27]]. The BPI-SF average pain item was responsive in a single phase 3 registration study supporting approval of abiraterone for patients with mCRPC [[27]]. The FACT-P was also responsive in both phase 3 registration studies for abiraterone and in phase 3 studies for enzalutamide and docetaxel for patients with mCRPC [[7],[26],[28],[29]]. However, based on the studies reviewed, responsiveness was not demonstrated for the PPI. Specifically, pain progression and pain response endpoints did not differentiate between treatment arms in the phase 3 registration study for cabazitaxel in patients with mCRPC [[30]]. Thus, responsiveness is strongest for the BPI-SF worst pain item and FACT-P followed by the BPI-SF average pain item in clinical trials evaluating recently approved treatments for patients with mCRPC.

#### Interpretation

Because statistical significance may be achieved with small changes in PRO measures, understanding what constitutes a clinically meaningful change on a PRO measure can facilitate interpretation of clinical trial results for treatment of mCRPC. Among the PRO measures reviewed, published information on the score interpretation in patients with mCRPC was identified for the BPI-SF worst pain item and FACT-P but not for the BPI-SF average pain item or PPI. Based on treatment-blinded data from 464 patients with mCRPC collected as part of a multinational phase 3 clinical trial, Regnault and colleagues confirmed that patient scores ≥ 5 on the BPI-SF worst pain item are associated with significant and meaningful impairments in patients with mCRPC, thus supporting the adequacy of this cut point as an appropriate definition of pain progression in this population [[31]]. Cella and colleagues also conducted a study to determine clinically meaningful changes for the FACT-P [[32]]. By applying both anchor-based and distribution-based methods to data from 809 patients with mCRPC who participated in a phase 3 trial evaluating atrasentan, a clinically meaningful change of 6 to 10 was estimated for the FACT-P total score (score range: 0–156) [[32]].

## Discussion

### PRO label claims in the US and the EU for mCRPC products

This review provides a critical evaluation and comparison of PRO claims approved by the FDA and the EMA for five recently approved products for mCRPC. Some concordance was seen between the FDA and the EMA for pain claims granted to enzalutamide and abiraterone based on the BPI-SF worst pain item. Baseline BPI-SF worst pain item data were included in both the US labels and EU SmPCs for enzalutamide and abiraterone. This concordance in the acceptance of pain claims may be explained in part by the fact that patient-assessed core symptoms of a disease such as pain are well-accepted primary and secondary efficacy endpoints in registration trials, according to the EMA reflection paper on HRQOL measures [[14]]. Similarly, the FDA PRO guidance highlights the assessment of pain intensity using a single-item measure as an obvious way to measure the impact of treatment on pain [[10]]. Furthermore, the FDA guidance for industry on cancer clinical trial design cites symptoms as a direct efficacy endpoint that can be used to support product approval [[33]].

However, even for the generally accepted concept of pain, there was still some discordance between the FDA and the EMA in the claim language allowed for mCRPC products reviewed. The primary difference in claim language between the US label and the EU SmPC for abiraterone was that the US label focused on the opiate use change, which was supported by the delay in pain progression, whereas the EU SmPC mentioned the time to pain progression more directly. The EMA also granted additional claim language in the SmPC for abiraterone, including “time to pain degradation,” “proportion of patients with pain palliation,” and “proportion of patients with pain progression” based on the BPI-SF worst pain item. No further claims were granted in the US label for this product. This discordance may be explained in part by the FDA’s comments within the abiraterone DAP: “Other secondary endpoints measured but not listed and not evaluated in the review included proportion of patients experiencing pain palliation using BPI-SF and analgesic score and time to pain progression…Reasons for not including them in the review are as follows: only a portion (<50%) of patients had data for the endpoints not listed as key secondary endpoints; measuring the endpoints was less objective, and their regulatory acceptability had not been evaluated by the Agency in terms of reliability, validity, ability to detect change, and interpretability in the study patient population; no pre-specified plan for multiple comparisons adjustment; changes in these endpoints do not constitute a basis for marketing approval or disapproval of abiraterone acetate for the proposed indication” [[11]].

Notably, a recent communication prepared by the FDA highlighted the following challenges in pain palliation measurement in cancer clinical trials [[34]]:

 Pain intensity and analgesic use assessment tools must be demonstrated to be reliable, valid, and sensitive to changes over time, consistent with FDA PRO guidance criteria.

 Enrollment eligibility criteria should ensure that patients are experiencing pain that is attributable to cancer at baseline.

 The mode of data collection (e.g., paper, electronic, internet, interactive voice response system, or interviewer-administered) must demonstrate measurement properties in keeping with the PRO guidance principles.

 Optimal timing for pain and analgesic assessment is over several consecutive days (e.g., daily over the 7-day period prior to a scheduled study visit).

 A pain palliation responder should be defined using both pain and analgesic use criteria, incorporating an analysis of tumor response that will support evidence of pain palliation response.

 There is a risk of missing data and inadvertent unblinding

Basch and colleagues stress the critical importance of tracking analgesic use with a content-valid analgesic log to ensure that pain palliation observed is truly the result of the treatment being studied rather than the result of an increase in analgesic use [[34]]. Furthermore, the analgesic log should be administered using the same schedule and recall period (e.g., past 24 hours) as the pain intensity assessment during clinical studies evaluating treatment. FDA feedback on the abiraterone DAP and Basch and colleagues’ [[34]] publication regarding assessment of pain palliation in cancer clinical trials highlight the many factors considered by the FDA when considering pain claims for a new cancer product.

Although the EMA granted additional pain claims to abiraterone based on the BPI-SF average pain item and to cabazitaxel based on the PPI, similar claims for these products were not granted by the FDA for these PRO measures. Based on publicly available information provided in the DAP for cabazitaxel, the following comments were made by FDA reviewers during the end-of-phase-2 meeting (EOP2) in response to questions submitted by the sponsor regarding the PPI and pain assessment: “We recommend that you submit the final version of the PPI and the AS [analgesic score] in the exact format it is administered in your protocol with instructions on how the instrument will be administered, directions explaining how scores will be derived, and how the statistical analyses will be applied; open-label data are only appropriate for labeling if results are convincing and conclusive; pain intensity should be assessed at screening, and then continued eligibility by pain score should be verified at baseline (i.e. before randomization/dosing); pain intensity should then be recorded daily, over the duration of the trial. There should also be evidence of efficacy over the entire duration of treatment; assessment of the ‘worst pain’ will provide more reliable results than ‘average pain’ over 24 hours.” However, it should be noted that the EOP2 meeting for cabazitaxel took place in 2006 and does not appear to represent the most current thinking by the FDA regarding pain assessment. As discussed earlier by Basch and colleagues, daily collection of pain and analgesic assessment for 7 consecutive days prior to an office visit is recommended over continuous daily collection of pain [[34]]. This recommendation is likely due to higher patient burden and higher noncompliance with continuous daily collection over the length of the study period. Furthermore, the DAP for cabazitaxel indicated that the most frequent and most severe protocol violations for the phase 3 registration study were for missing pain assessments or analgesic scores, with less than 50% of each treatment group reporting analyzable data. Although similar explanations from FDA reviewers were not available in the abiraterone DAP, the FDA’s stated preference for the evaluation of “worst pain” over the past 24 hours within the cabazitaxel DAP may also partly explain why the claim was not granted for abiraterone based on the BPI-SF “average pain” item.

The largest area of discord identified between the EMA and the FDA was for HRQOL claims. HRQOL claims were granted by the EMA for both abiraterone (“time to degradation in FACT-P total score”) and radium Ra 223 dichloride (“Relative to placebo, the decline in quality of life was slower for Ra-223 during the on-treatment period as measured by EQ-5D utility index score [−0.040 versus −0.109; p = 0.001], EQ-5D self-reported VAS [−2.661 versus −5.860; p = 0.018], and the FACT-P total score [−3.880 versus −7.651, p = 0.006] but did not reach published minimally important differences. There is limited evidence that the delay in loss of HRQOL extends beyond the treatment period”). Similar HRQOL claims for these products were not granted by the FDA.

Comments by FDA reviewers found within the radium Ra 223 dichloride DAP may provide some insight into why a HRQOL claim was not granted by the FDA for this product. Specifically, the reviewer comments on the PRO results from the key pivotal trial for radium Ra 223 dichloride stated that “FACT-P and EQ-5D total scores showed a slight improvement for patients receiving Ra-223 when compared to placebo. When evaluated by visit, the statistical significance in the difference between groups decreased over time. Whether this was due to the loss of anti-cancer effect of Ra-223 with time or was due to decreased data completion rates is not known, although both are likely contributors. The FDA SEALD cites limitations specific to the current Ra-223 application including small number of assessments (maximum of 4 for FACT-P), low rate of completion at week 24+ as well as a small observed magnitude of treatment effect. Despite the limitations in choice of instrument, frequency of assessments and completeness of data, the quality-of-life results are supportive of the overall application in that, on average, the available data appear to trend toward an improvement in the Ra-223 arm and do not show a detriment to quality of life measures or pain in patients treated with Ra-223 when compared to placebo.” Similar comments from FDA reviewers were not available within the abiraterone DAP to further explain why HRQOL claims were not granted for this product.

As demonstrated by the SmPC language for both cabazitaxel and radium Ra 223 dichloride, the EMA provided more comprehensive coverage of both the positive and negative PRO outcomes from the registration studies for these products, whereas negative PRO outcomes were discussed within the DAP but did not translate into label language in the US.

The results of our research on PRO label claims for mCRPC products recently approved in the US and the EU further support the conclusions from several earlier studies with similar objectives [[10],[35]-[37]]. Demuro and colleagues’ comparison of PRO label claims granted from 2006 to 2010 for new drug entities or biologic licensed agents by the FDA and the EMA indicated that the EMA is more likely than the FDA to grant PRO claims and typically does so for higher-order constructs (e.g., HRQOL), whereas PRO claims in the US are most often limited to those based on symptom improvement [[19]]. Similar findings also resulted from earlier research by Coombs and colleagues when PRO claims in the US and the EU were compared for oncology products [[37]].

### Evaluation of instrument properties for selected PRO measures against FDA guidance criteria

Of the measures evaluated, the BPI-SF worst pain item has the strongest measurement properties in patients with mCRPC. Most importantly, content validity has been established in this population, a key criterion necessary for FDA acceptance in supporting labeling claims. Furthermore, although evidence of known-groups validity was not identified for this measure, test-retest reliability, construct validity, and responsiveness have all been established in patients with mCRPC [[27],[34]]. Other key strengths of this measure include the brief (24-hour) recall period; the single-item assessment (low burden on patients to complete); the 11-point numeric rating scale, which is well-accepted by patients, clinicians, and the FDA; and information available to assist interpretation of clinical trial results in patients with mCRPC with bone metastases [[31]]. Based on this evidence and the ability to achieve successful labeling claims of “delay in pain progression” for enzalutamide and abiraterone in both the US and the EU, as well as additional claims in the EU for “time to pain degradation,” “proportion of patients with pain palliation,” and “proportion of patients with pain progression,” the BPI-SF worst pain item is recommended for future evaluation of pain in trials evaluating treatment for patients with mCRPC and for pursuit of similar labeling claims in both the US and the EU.

The identified evidence in support of the BPI-SF average pain item is weaker when compared with FDA PRO guidance criteria. Without documented evidence of content validity in patients with mCRPC, the FDA is unlikely to grant PRO claims based on this single item. However, the BPI-SF average pain item is recommended for publications supporting future FDA-approved products for mCRPC and may achieve EMA-approved SmPC claims if the data are convincing.

Of the three pain measures evaluated, the PPI component of the MPQ has the least evidence to support its future use in clinical trials evaluating mCRPC treatments. When considering evaluation of pain in patients with mCRPC and if the desire is to achieve a product label claim in the US or the EU, the PPI is not recommended.

The FACT-P was the only HRQOL measure evaluated against FDA PRO guidance criteria. Key strengths for the FACT-P are established reliability (test-retest and internal consistency), construct validity, known-groups validity, responsiveness, and established definition for clinically meaningful changes to assist with interpretation in clinical trials evaluating patients with mCRPC [[32]]. In particular, the FACT-P was responsive in both phase 3 registration studies for abiraterone as well as phase 3 studies for enzalutamide and docetaxel for patients with mCRPC but was less responsive in the registration study for radium Ra 223 dichloride described in the SmPC [[7],[28],[29],[34]]. Based on further examination of the two EMA-approved SmPCs that included FACT-P claims, there was clear positive claim language favoring abiraterone in the SmPC for “risk of FACT-P total score degradation” and “time to degradation in FACT-P total score,” whereas the analysis of change from baseline in registration studies for radium Ra 223 dichloride resulted in an SmPC claim that scores on this measure did not reach the published minimum important difference. Given the limitation on content validity for the FACT-P in patients with mCRPC, this measure is not recommended to support US labeling claims for future products. Because the FACT-P is validated and clearly very responsive in studies evaluating treatment for mCRPC, the FACT-P should still be considered for the evaluation of PC-specific HRQOL to provide data for US publication and support of SmPC claims in the EU.

More recently, the National Comprehensive Cancer Network FACT Prostate Symptom Index-17 (NCCN-FACT FPSI-17), a new prostate symptom index, was developed based on qualitative input from patients with advanced (stage 3 and 4) castration-resistant PC, which can be used to examine the effectiveness of noncurative treatments in advanced PC [[38]]. Although the NCCN-FACT FPSI-17 appears to have initial evidence of content validity, reliability, and construct validity in patients with mCRPC, publications documenting its responsiveness in studies evaluating treatment for patients with mCRPC are currently not available [[38]]. Once additional studies are completed to further validate this measure and establish responsiveness, it may potentially be considered for inclusion in future studies for treatment of mCRPC designed to obtain US labeling claims focused on PC symptom improvement, provided all FDA PRO guidance criteria beyond content validity are also sufficiently met in this population.

Notably, the concept of fatigue, a prevalent disease- and treatment-related symptom of patients with mCRPC, was measured using a fatigue-specific PRO measure (Brief Fatigue Inventory) in registration trials for both abiraterone and enzalutamide based on information provided in each product’s DAP and EPAR [[4]]. However, neither the FDA nor the EMA granted a fatigue claim. This finding is not surprising given the recent views on fatigue assessment expressed by an FDA representative that fatigue is a multidomain concept not measurable with a single item; it is believed that patients do not use the term “fatigue”; problems with instrument content validity have not allowed conclusion of benefit; and, finally, a clear link between fatigue and the disease or treatment has not been found [[39]]. To address these concerns, a consortium research project entitled Patient-Reported Outcomes of Fatigue–Cancer has been established “to define cancer related fatigue and determine how it should be measured from a patient perspective” [[40]]. The Cancer Fatigue–Symptom Severity Assessment, a new multidimensional fatigue measure, has been developed out of this consortium, is currently undergoing psychometric validation, and is planned for drug development tool qualification by the FDA [[40]]. This measure may eventually be accepted by the FDA and the EMA to support fatigue-specific PRO labeling claims for new cancer products.

There are some notable limitations to our research. First, although this study included the majority of recently approved products for mCRPC, not all mCRPC products were included [[10]]. Second, any PRO measures included in clinical trials for mCRPC products reviewed and mentioned in the DAP or EPAR for a product but not resulting in US label or EU SmPC claims were excluded from this review. Third, our review of regulatory feedback during the approval process for each of the products was limited to the information that was made publicly available on the FDA and EMA websites. The drug manufacturers may possess additional proprietary information that was not included. Finally, the nature of our literature searches to identify the desired information supporting each PRO measure of interest was targeted rather than systematic. Thus, it is possible that other information exists either to support or refute the information presented in this paper.

## Conclusions

Our research findings can be used to inform the development of a PRO strategy for phase 2 and 3 clinical trials designed to support a product label claim for a new mCRPC treatment. The BPI-SF worst pain item is recommended for use in combination with analgesic use assessment to evaluate pain progression and pain palliation. Based on the demonstrated content validity in patients with mCRPC and documented measurement properties in this population, as well as the recent claims achieved for enzalutamide and abiraterone, the BPI-SF worst pain item alone may be supportive of future PRO-related claims in both the US and the EU.

Assessment of PC-specific HRQOL using the FACT-P to achieve time to FACT-P total score degradation claims may continue to be accepted by the EMA and incorporated into EU SmPCs, whereas FACT-P data are recommended only for publication purposes in the US. An mCRPC symptom index meeting all FDA PRO guidance criteria, including content validity, likely has a greater chance of supporting both US label and EMA SmPC claims. The EQ-5D is recommended for PRO strategy development for any new mCRPC treatment to identify health utility values for comparison across diseases. EQ-5D with VAS data may also be used to support SmPC claims in the EU; however, in the US, these data are recommended for publication rather than label claims. Finally, fatigue is another important disease- and symptom-related concept in patients with mCRPC that should be considered for assessment in future clinical trials using a validated assessment. Further work is needed in this area to increase the likelihood of successful fatigue-specific product label claims for mCRPC products in the US and the EU.

## Abbreviations

AS: Analgesic score

BPI: Brief pain inventory

BPI-SF: Brief pain inventory–short form

DAP: Drug approval package

EQ-5D: EuroQol 5 dimensions

EMA: European medicines agency

EOP2: End-of-phase-2 meeting

EPAR: European public assessment report

EQ-5D: EuroQoL 5 dimensions

EU: European Union

FACT-G: Functional assessment of cancer therapy–general

FACT-P: Functional assessment of cancer therapy–prostate module

FDA: Food and drug administration

HCP: Health care professional

HRQOL: Health-related quality of life

ICC: Intraclass correlation coefficient

mCRPC: Metastatic castration-resistant prostate cancer

MPQ: McGill pain questionnaire

NCCN-FACT FPSI-17: National comprehensive cancer network FACT prostate symptom index-17

NRS: Numeric rating scale

PC: Prostate cancer

PPI: Present pain intensity

PRO: Patient-reported outcome

PROQOLID: Patient-reported outcome quality of life instrument database

RTI-HS: RTI health solutions

SEALD: Study endpoints and label development

SmPC: Summary of product characteristics

US: United States

VAS: Visual analogue scale

VRS: Verbal rating scale

## Competing interests

Funding for this study was provided by Boehringer Ingelheim GmbH.

At the time of this study, Marci Clark, Nimanee Harris, and Catherine Copley-Merriman were employed by RTI Health Solutions, which provides research and consulting services to help biopharmaceutical companies successfully develop and gain market approval for their products. As salaried employees, Dr. Clark, Ms. Harris, and Ms. Copley-Merriman work with various companies but do not receive direct payment or honoraria from them for services rendered. Dagmar Kaschinski and Ingolf Griebsch are employees of Boehringer Ingelheim GmbH.

## Authors’ contributions

IG and DK had the idea for the study. MC oversaw its design and contributed to the data collection in collaboration with NH. MC did all the analyses and drafted the paper. IG, DK, MC, NH, and CCM contributed to the design of the manuscript, interpretation of results, and discussion of the findings. All authors read and approved the final manuscript.
